# Circulating bioactive bacterial DNA is associated with immune activation and complications in common variable immunodeficiency

**DOI:** 10.1172/jci.insight.144777

**Published:** 2021-10-08

**Authors:** Hsi-en Ho, Lin Radigan, Gerold Bongers, Ahmed El-Shamy, Charlotte Cunningham-Rundles

**Affiliations:** 1 Department of Medicine and; 2 Microbiome Translational Center, Precision Immunology Institute, Department of Oncological Sciences, Icahn School of Medicine at Mount Sinai, New York, New York, USA.

**Keywords:** Immunology, Inflammation, Beta cells, Genetic diseases, Immunoglobulins

## Abstract

Common variable immunodeficiency (CVID) is characterized by profound primary antibody defects and frequent infections, yet autoimmune/inflammatory complications of unclear origin occur in 50% of individuals and lead to increased mortality. Here, we show that circulating bacterial 16S rDNA belonging to gut commensals was significantly increased in CVID serum (*P* < 0.0001), especially in patients with inflammatory manifestations (*P* = 0.0007). Levels of serum bacterial DNA were associated with parameters of systemic immune activation, increased serum IFN-**γ**, and the lowest numbers of isotype-switched memory B cells. Bacterial DNA was bioactive in vitro and induced robust host IFN-**γ** responses, especially among patients with CVID with inflammatory manifestations. Patients with X-linked agammaglobulinemia (Bruton tyrosine kinase [BTK] deficiency) also had increased circulating bacterial 16S rDNA but did not exhibit prominent immune activation, suggesting that BTK may be a host modifier, dampening immune responses to microbial translocation. These data reveal a mechanism for chronic immune activation in CVID and potential therapeutic strategies to modify the clinical outcomes of this disease.

## Introduction

Common variable immunodeficiency (CVID), the most prevalent symptomatic primary immunodeficiency, is characterized by low levels of serum IgG, IgA, and/or IgM, and a lack of production of specific antibodies (1–3). As with other primary immune defects, patients with CVID are susceptible to recurrent severe infections. Notably, however, at least 50% of patients with CVID develop additional inflammatory complications (4, 5). These noninfectious manifestations include autoimmunity, interstitial lung disease, enteropathy, nodular regenerative hyperplasia of the liver, systemic granulomatous disease, lymphoid hyperplasia, and lymphoid malignancy (4–7). These complications are a major clinical challenge because they are not substantially ameliorated by standard IgG replacement therapy. As a whole, inflammatory conditions lead to an estimated 11-fold increased morbidity and mortality in patients with CVID (5).

Recent investigations have identified genetic defects leading to loss of B cell development and other defects in immune regulation in 20%–25% of individuals, but the pathogenesis of inflammatory complications in CVID has remained unexplained in most cases (8, 9). We previously demonstrated marked upregulation of IFN-related pathways in these individuals with CVID by mRNA transcriptional profiling (10). This IFN signature also distinguished individuals with CVID who had inflammatory conditions from other individuals with CVID and from healthy controls. To identify the origin of this pathological IFN signature, we used mass cytometry and found an expanded population of innate lymphoid cells (ILCs) positive for IFN-γ, IL-17A, and IL-22 in the peripheral blood and gastrointestinal and lung tissues of individuals with CVID who had inflammatory complications (11). ILCs typically play an important role in host-commensal homeostasis (12), and their excessive activity and/or proliferation appear to contribute to systemic and organ-specific inflammation in CVID. However, the stimulus underlying these immune responses has remained unknown.

Humoral immunity contributes to anatomical containment of commensal organisms in experimental animal models (13). In mice, secretory IgA and IgM have been shown to limit bacterial translocation from mucosal compartments (14–17). Microbiota-specific IgG has been detected in mice and healthy humans, with evidence of direct binding to a subset of gut microbiota (18–20). When commensal organisms are not appropriately contained, bacterial translocation, defined as the translocation of bacteria and/or their products without overt bacteremia, has been shown to drive chronic inflammation in disease states, such as HIV and inflammatory bowel disease (21, 22). In this study, we evaluated the functional impact of primary antibody defects on host-commensal compartmentalization in patients with lack of antibody production. Specifically, we sought the presence of a conserved DNA sequence encoding bacterial 16S RNA (16S rDNA) in the serum of a large cohort of well-characterized patients with CVID and for comparison, patients with X-linked agammaglobulinemia (XLA). We also asked whether the circulating bacterial DNA could be a driver of inflammatory complications in CVID, serving as a critical immune stimulus for the pathological IFN-γ signature found in inflammatory CVID.

## Results

### Raised circulating bacterial DNA in CVID and XLA.

**Figure 1:**
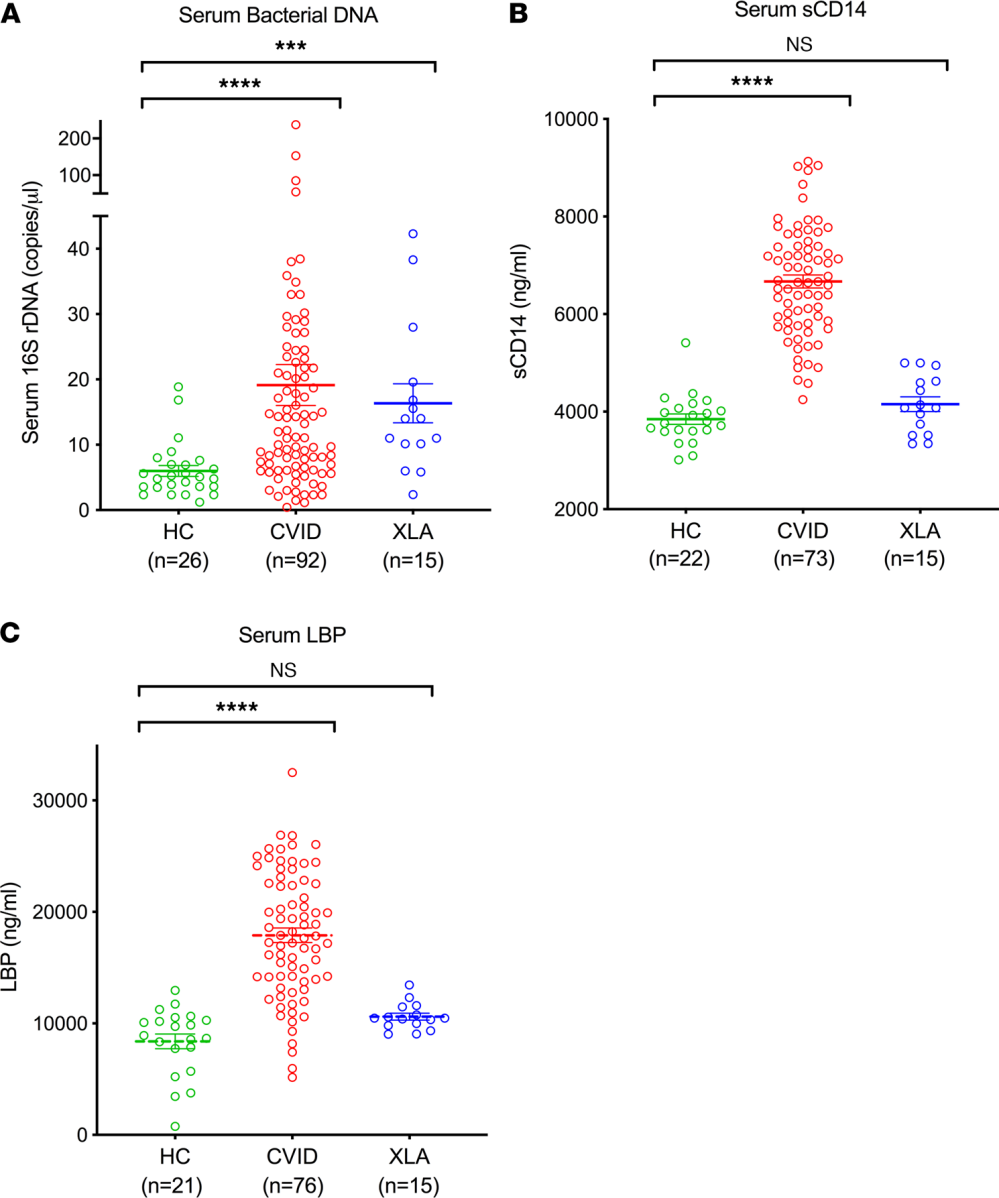
Serum circulating bacterial DNA, sCD14, and LBP levels in CVID as compared with XLA and healthy individuals. (**A**) Serum 16S rDNA, (**B**) serum sCD14, and (**C**) serum LBP levels in healthy controls (HCs), patients with CVID, and patients with XLA. Kruskal-Wallis test revealed significant differences between groups for bacterial 16S rDNA (*P* < 0.0001); 1-way ANOVA revealed significant differences between groups for sCD14 (*P* < 0.0001) and LBP (*P* < 0.0001). The data are expressed as the mean ± SEM. ***P < 0.001, ****P < 0.0001 by Kruskal-Wallis with Dunn’s multiple-comparison post hoc test (**A**) and 1-way ANOVA with Tukey’s post hoc test (**B** and **C**). NS, not significant.

To assess the presence and magnitude of microbial translocation in CVID, we first employed quantitative PCR to measure bacterial 16S rDNA levels in serum samples obtained from 92 patients with CVID in their usual state of health and 26 healthy individuals. We also measured serum 16S rDNA levels from 15 patients with XLA to evaluate whether microbial translocation was a shared feature among patients with profound defects in humoral immunity (Table 1). Bacterial 16S rDNA levels were significantly elevated in CVID sera (mean 19.15 copies/L, range 0.4–237.4 copies/L) as compared with healthy controls (mean 5.99 copies/L, range 1.2–18.88 copies/L, *P* < 0.0001). Patients with XLA also had similarly elevated serum 16S rDNA levels as compared with healthy controls (mean 16.34 copies/L, range 2.36–42.31 copies/L, *P* = 0.0008) (Figure 1A). The size of bacterial 16S rDNA amplicons derived from the serum samples was approximately 254 nucleotides long, providing a minimal estimate of the circulating bacterial DNA size in the patients.

### Increased serum-soluble CD14 and lipopolysaccharide-binding protein in CVID but not in XLA.

We next assessed whether bacterial translocation, identified as elevated circulating bacterial DNA, was accompanied by systemic immune activation in CVID or XLA. We quantified serum-soluble CD14 (sCD14), which is secreted by monocytes/macrophages upon exposure to bacterial products, including CpG DNA, Gram-negative LPS, and Gram-positive bacterial components (23–25). We also measured serum lipopolysaccharide-binding protein (LBP), produced by hepatocytes in response to LPS stimulation (26). As compared with healthy controls, serum sCD14 levels were significantly increased in patients with CVID (mean 6670 ng/mL vs. 3846 ng/mL, *P* < 0.0001, Figure 1B). Similarly, serum LBP was significantly elevated in CVID compared with healthy controls (mean 17,906 ng/mL [CVID] vs. 8392 ng/mL [healthy controls], *P* < 0.0001, Figure 1C). Among patients with CVID, serum sCD14 was positively associated with serum bacterial 16S rDNA (Spearman’s *r* = 0.28, *P* = 0.0166, Supplemental Figure 1A; supplemental material available online with this article; https://doi.org/10.1172/jci.insight.144777DS1). There was also a positive association between sCD14 and LBP (Spearman’s *r* = 0.21, *P* = 0.0735, Supplemental Figure 1B). However, in contrast to CVID, these systemic markers of immune activation were not significantly elevated in patients with XLA as compared with healthy controls (Figure 1, B and C).

### Lack of detection of endotoxin in CVID serum.

Endotoxin from Gram-negative bacteria has been reported in serum in a few studies of patients with CVID but not found in other studies (27–30). Confirming the latter reports, we did not detect endotoxin in the serum of our CVID cohort in either undiluted and diluted serum using 4 commercial assays (EndoLISA, Pierce LAL Chromogenic Endotoxin Quantitation kit, Limulus Amebocyte Lysate Chromogenic Endpoint assay, and ToxinSensor). Given that 90 out of 92 patients with CVID in this cohort were on IgG replacement therapy, we tested whether endotoxins could perhaps be bound by the infused IgG. By examining the binding specificity of i.v. Ig products, we found that the polyclonal IgG antibodies contained in these commercial products bound endotoxin in a dose-dependent manner, suggesting potential interference either within serum samples themselves and/or in the context of the detecting assays (Supplemental Figure 2).

### Markers of mucosal epithelial barrier dysfunction in CVID.

**Figure 2:**
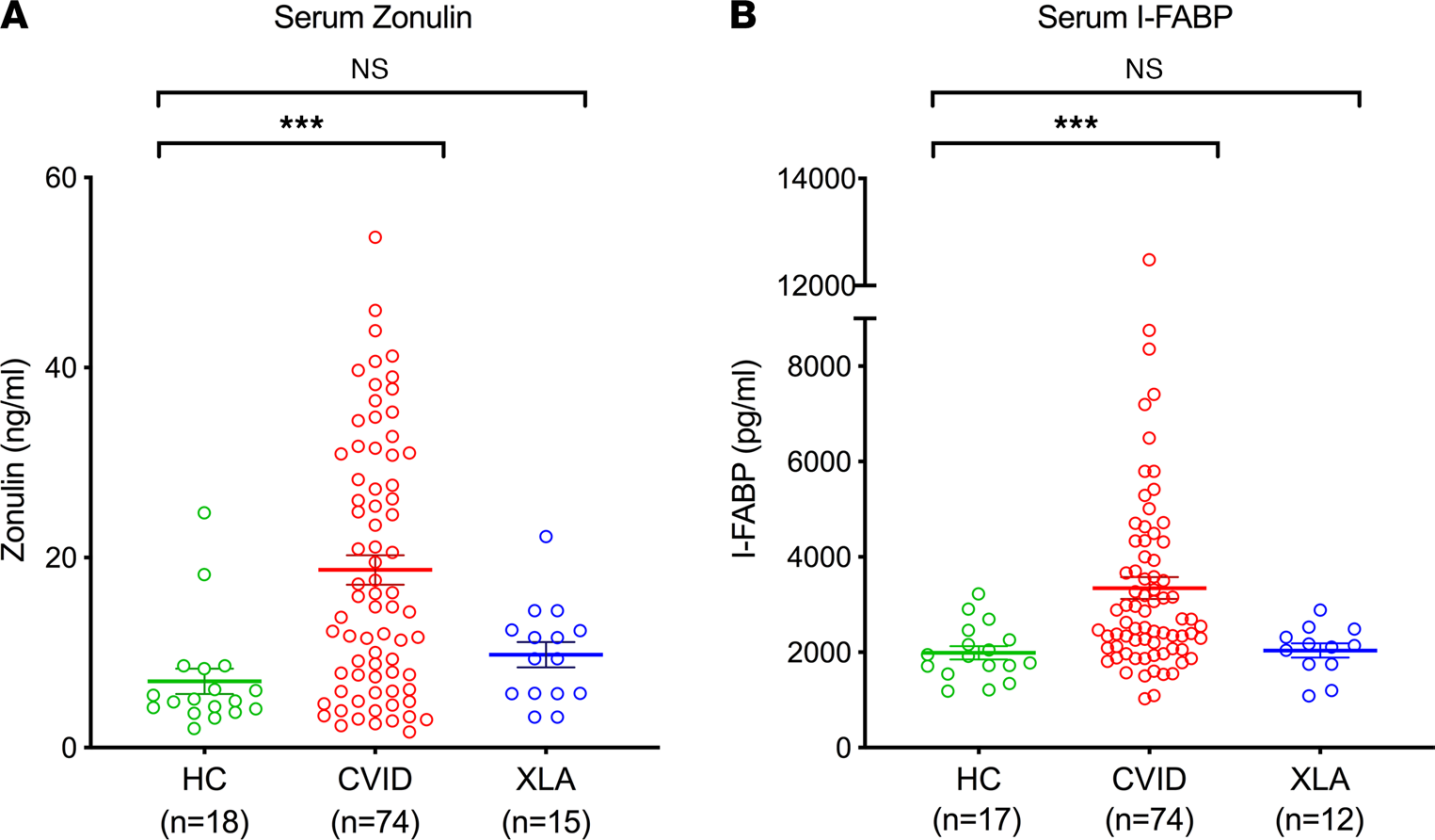
Mucosal epithelial barrier dysfunction in CVID. (**A**) Serum zonulin and (**B**) I-FABP levels in healthy controls (HCs), patients with CVID, and patients with XLA. Kruskal-Wallis test revealed significant differences between groups for zonulin (*P* = 0.0003) and I-FABP (*P* = 0.0002). The data are expressed as the mean ± SEM. ***P < 0.001 by Kruskal-Wallis with Dunn’s multiple-comparison post hoc test. NS, not significant.

Next, we sought evidence of a dysfunctional mucosal barrier that, in addition to the loss of mucosal antibody, could contribute to the translocation of microbial products in CVID. Zonulin is a human protein positively associated with intestinal wall permeability through its role as modulator of intercellular tight junctions between epithelial cells (31–33). Zonulin levels in CVID serum were markedly elevated as compared with healthy controls (mean 18.71 ng/mL vs. 6.99 ng/mL, respectively, *P* = 0.0003, Figure 2A). Additionally, we evaluated serum levels of intestinal fatty-acid binding protein (I-FABP), an intestinal epithelium-specific protein that can leak into circulation in the setting of gut barrier dysfunction (34, 35). Here also, serum I-FABP levels were significantly elevated among patients with CVID compared with healthy controls (mean 3346 pg/mL vs. 1992 pg/mL, respectively, *P* = 0.0006, Figure 2B). In contrast to the patients with CVID, neither serum zonulin nor I-FABP levels were significantly elevated among patients with XLA (Figure 2, A and B). When examining the clinical phenotypes of the CVID cohort, we found that serum zonulin and I-FABP levels were significantly elevated among patients with CVID regardless of the presence or absence of known enteropathy (Supplemental Figure 3, A and B).

### The gastrointestinal tract is a major source of bacterial translocation in CVID.

**Figure 3:**
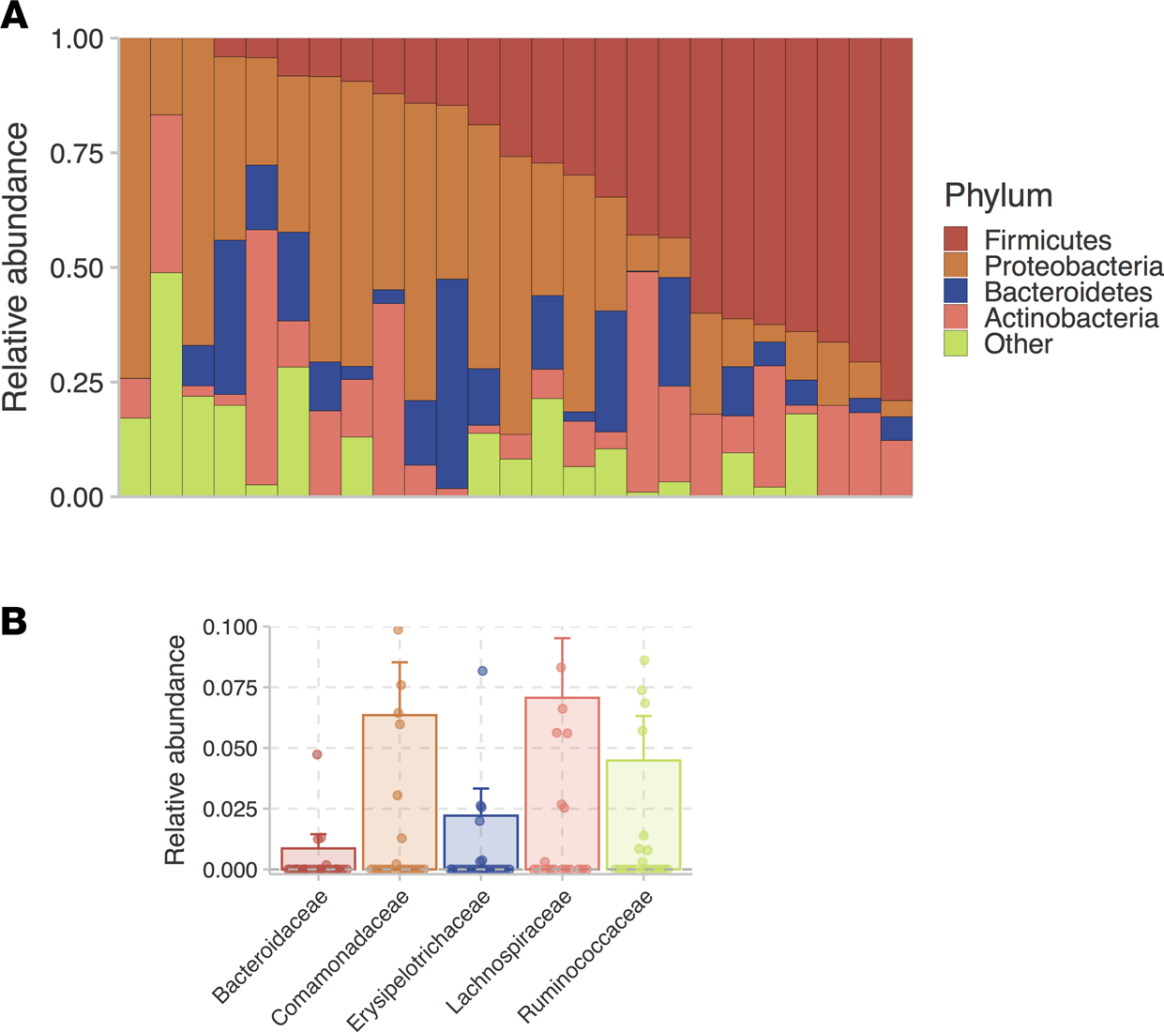
16S profiling of circulating bacterial DNA in CVID identified genetic materials belonging to gut-associated commensals and an abundance of bacterial families that are normally highly coated by secretory IgA/IgM. (**A**) Relative abundances of bacterial phyla detected in the sera of patients with CVID (*n* = 25). Each column represents serum from a patient with CVID. (**B**) Most abundant bacterial families detected in the sera of patients with CVID (*n* = 25). The data are expressed as the mean relative abundance ± SEM.

The gastrointestinal tract contains a large bacterial mass (36). We hypothesized that the gastrointestinal tract could be a principal source of bacterial translocation in antibody deficiency. To evaluate for this, we sequenced circulating 16S rRNA genes (rDNA) to identify the corresponding bacterial taxa. Serum 16S rRNA gene profiling of serum of 25 patients with CVID revealed that the translocation of bacterial DNA was derived from a diverse range of commensal species, predominantly by gut-associated bacterial phyla, including Firmicutes, Bacteroidetes, Actinobacteria, and Proteobacteria (Figure 3A). Notably, DNA from gut commensal families previously reported to be highly coated by secreted antibodies (*Lachnospiraceae, Ruminococcaceae, Erysipelotrichaceae, Bacteroidaceae*, and *Comamonadaceae*) in healthy humans were found in high abundance in the sera of these patients with CVID (Figure 3B) (37–40). All bacterial families detected and ranked by taxonomic abundance are tabulated in Supplemental Table 1.

### Translocation of bacterial DNA is associated with inflammatory manifestations in CVID.

**Figure 4:**
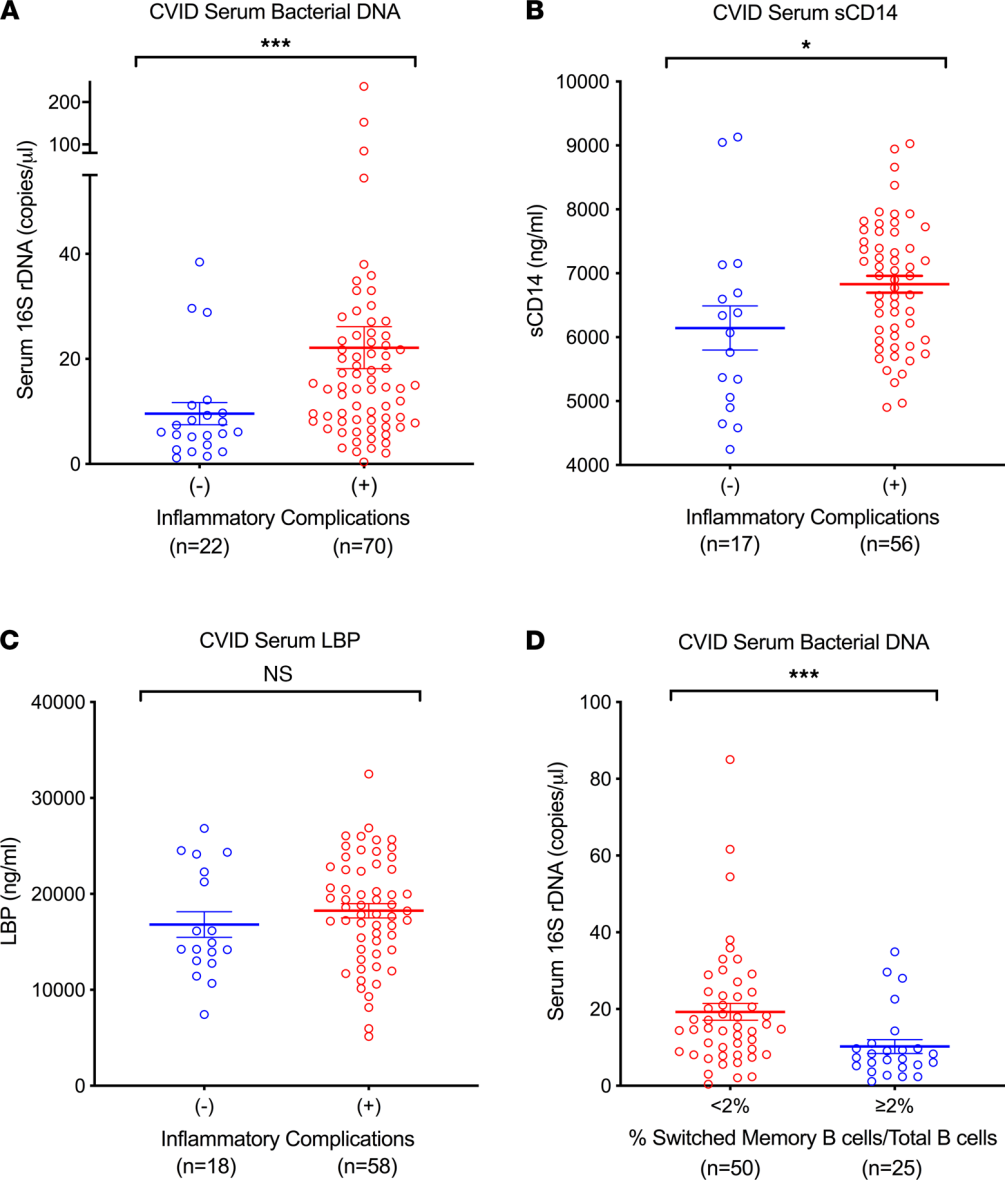
Circulating bacterial DNA is related to inflammatory manifestation in CVID. Comparison of (**A**) serum 16S rDNA, (**B**) serum sCD14, and (**C**) LBP levels in patients with CVID with and without autoimmune/inflammatory complications. Mann-Whitney *U* test revealed significant differences between groups for bacterial 16S rDNA (*P* = 0.0007). Unpaired *t* test revealed significant differences between groups for sCD14 (*P* = 0.027). (**D**) Comparison of serum 16S rDNA levels in patients with CVID with less than 2% versus 2% or more of CD19^+^CD27^+^IgM^–^IgD^–^ switched memory B cells/CD19^+^ B cells. Mann-Whitney *U* test revealed significant differences between groups for bacterial 16S rDNA (*P* = 0.0008). The data are expressed as the mean ± SEM. *P < 0.05, ***P < 0.001 by Mann-Whitney *U* test (**A** and **D**) and unpaired *t* test (**B**). NS, not significant.

We then asked whether bacterial translocation, specifically higher levels of serum bacterial 16S rDNA levels, was associated with the inflammatory manifestations in CVID. Indeed, patients with CVID with inflammatory complications had significantly higher serum bacterial DNA levels (mean 22.15 copies/L, range 0.4–237.4 copies/L) as compared with CVID individuals without such conditions (mean 9.59 copies/L, range 1.14–38.46 copies/L, *P* = 0.0007, Figure 4A). Serum sCD14 levels were also significantly elevated in patients with CVID who had inflammatory complications compared with those without such complications (mean 6829 ng/mL vs. 6144 ng/mL, respectively, *P* = 0.027, Figure 4B) but the differences were smaller. The mean serum LBP levels were also higher in those with inflammatory manifestations, though this did not reach statistical significance (Figure 4C).

### Bacterial translocation and immunological parameters.

We then evaluated whether specific host immune parameters were associated with elevated circulating bacterial DNA. First, bacterial 16S rDNA levels were not correlated with blood IgG levels in CVID or patients with XLA, either at diagnosis (baseline) or with Ig replacement (not shown). In this CVID cohort, where serum IgA and IgM were severely reduced in most patients, we found that serum 16S rDNA was also not related to serum IgA or IgM levels (not shown). However, for patients with CVID, those with severely reduced isotype-switched memory B cells (defined as <2% of total CD19^+^B cells) (41) had significantly higher serum bacterial 16S rDNA levels as compared with those with higher numbers of these cells (mean 19.27 copies/L, range 0.4–85 copies/L vs. mean 10.24 copies/L, range 1.14–34.9 copies/L, respectively, *P* = 0.0008, Figure 4D). Overall, there was a negative correlation between percentages of isotype-switched memory B cells and circulating bacterial DNA levels (Supplemental Figure 4). There was no correlation between bacterial 16S rDNA levels and circulating CD3^+^, CD4^+^, or CD8^+^ T cell counts (not shown). In our cohort, we also did not identify serum bacterial DNA differences among the subset of 16 patients with known monogenic defects (*TNFRSF13B*, *n* = 7; *CTLA4*, *n* = 3; *STAT3* gain of function, *n* = 2; *IKZF1*, *KMT2D*, *NFKB1*, *PIK3CA*, *n* = 1 each; Supplemental Figure 5).

### Bacterial DNA as an immune stimulant for IFN-γ production in CVID.

**Figure 5:**
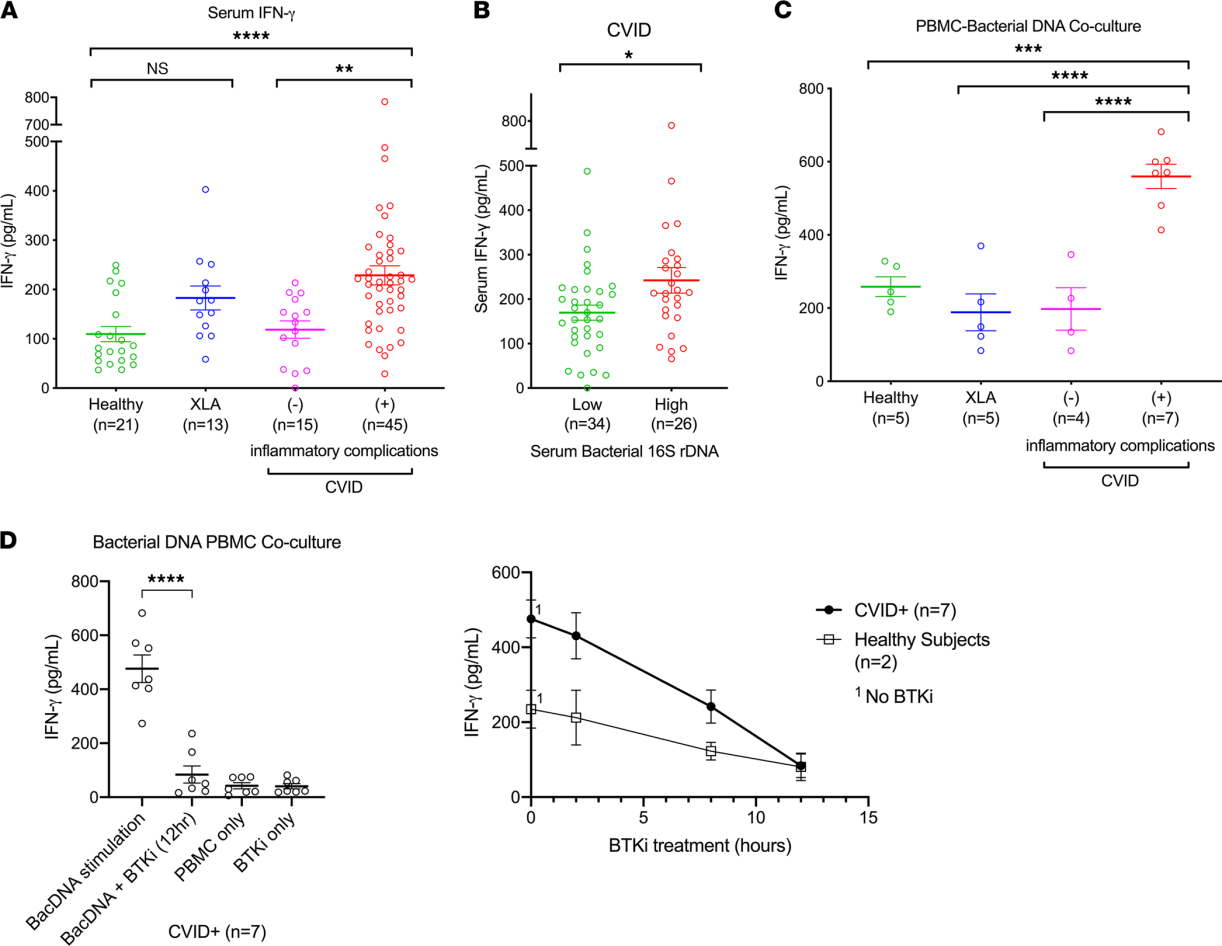
Bacterial DNA is an immune stimulant for IFN-γ production in CVID. (**A**) Serum IFN-γ levels in healthy controls (HCs), patients with XLA, and patients with CVID with or without autoimmune/inflammatory complications (CVID+, CVID, respectively). One-way ANOVA revealed significant differences between groups (*P* < 0.0001) (**B**) Serum IFN-γ levels in patients with CVID with low versus high serum bacterial 16S rDNA. High serum bacterial 16S rDNA is defined as levels higher than the 90th percentile of healthy controls. Unpaired *t* test revealed significant differences between groups (*P* = 0.0258) (**C**) IFN-γ levels in the culture supernatants of PBMCs isolated from HCs and XLA, CVID, and CVID+ patients on day 3 after coculture with bacterial DNA (7.5 pg/mL). One-way ANOVA revealed significant differences between groups (*P* < 0.0001). (**D**) IFN-γ levels in the culture supernatants of PBMCs isolated from CVID+ patients on day 3 after coculture with bacterial DNA (7.5 pg/mL), with or without a BTK inhibitor (BTKi; PCI 29732, 0.5 nM; left panel). Unpaired *t* test revealed significant differences between groups (*P* < 0.0001). Bacterial DNA-induced IFN-γ levels in response to BTKi treatment over time (PCI 29732, 0.5 nM; 0–12 hours) in PBMCs isolated from CVID+ and healthy individuals (right panel). The data are expressed as the mean ± SEM. *P < 0.05, **P < 0.01, ***P < 0.001, ****P < 0.0001 by 1-way ANOVA with Tukey’s post hoc test (**A** and **C**) and unpaired *t* test (**B **and** D**). NS, not significant.

We previously showed that marked upregulation of IFN-related pathways distinguished patients with CVID with inflammatory conditions from those without and that patients with inflammatory CVID had detectable serum IFN-γ (10, 11). This was confirmed here: serum IFN-γ levels were significantly elevated in these individuals with CVID when compared with other patients with CVID, patients with XLA, and healthy controls (mean 228.6 pg/mL, 118.6 pg/mL, 182.8 pg/mL, and 109.5 pg/mL, respectively, *P* < 0.0001, Figure 5A). IFN-γ levels were also modestly elevated in XLA, but this was not significant when compared with healthy controls (Figure 5A).

Next, we sought to determine whether circulating bacterial DNA might be bioactive and induce significant IFN-γ secretion in CVID. In vivo, we found that patients with CVID with high serum bacterial DNA (defined as >90th percentile of healthy controls) had significantly higher same-day serum IFN-γ levels compared with those with lower serum 16S rDNA (mean IFN-γ 242.3 pg/mL vs. 169.7 pg/mL, respectively, *P* = 0.0258, Figure 5B). To further investigate this, we cultured PBMCs isolated from patients with CVID and controls with the 16S bacterial DNA standard and measured IFN-γ production. Bacterial DNA effectively induced IFN-γ production in all study participants, but it induced significantly higher IFN-γ secretion in patients with CVID who had inflammatory conditions, as compared with other patients with CVID, patients with XLA, and healthy controls (mean 560.0 pg/mL, 197.7 pg/mL, 188.5 pg/mL, and 258.6 pg/mL, respectively, *P* < 0.0001, Figure 5C) in a dose-dependent manner (Supplemental Figure 6). The digestion of bacterial DNA by DNase abrogated the IFN-γ response in the PBMC coculture, demonstrating the specificity of this response (Supplemental Figure 7). Separately, we asked whether the presence of human cell-free DNA might enhance or inhibit the observed IFN-γ response among patients with CVID in this assay: no changes in IFN-γ response were observed (data not shown).

### B cell numbers, Bruton tyrosine kinase, and IFN-γ production.

Given the differential response between CVID and patients with XLA, we next examined the role of B cells in this bacterial-driven response. However, there was no significant correlation between peripheral B cell numbers and serum IFN-γ levels in vivo in patients with CVID (Supplemental Figure 8). Similarly, we did not find that the in vitro bacterial DNA–stimulated IFN-γ response among the very rare patients with CVID who had inflammatory complications with an absence of peripheral B cells was significantly different from those with peripheral B cells (mean IFN-γ: 555.6 pg/mL [*n* = 3] vs. 563.3 pg/mL [*n* = 4], respectively). We then investigated whether the loss of Bruton tyrosine kinase (BTK) signals in XLA could lead to the differential IFN-γ response to bacterial stimuli between patients with CVID and patients with XLA. To answer this question, we examined the effect of BTK inhibition (42) in PBMC cultures of patients with CVID and inflammatory complications. Indeed, we found that BTK inhibition significantly reduced the IFN-γ response to bacterial DNA stimulation in these patients with CVID (mean IFN-γ 476.0 pg/mL [bacterial DNA stimulation] vs. 83.9 pg/mL [bacterial DNA stimulation + BTK inhibition], *P* < 0.0001, Figure 5D).

Taken together, these results indicate that circulating bacterial DNA is bioactive and capable of driving systemic immune activation in susceptible hosts and, in particular, the IFN-γ signature we previously observed in patients with CVID and inflammatory complications.

## Discussion

The compartmentalization of host and commensals is essential for health, preventing microbial-driven inflammation and translocation. In this study, we demonstrated direct evidence of bacterial translocation from mucosal surfaces into the systemic circulation (16S rDNA) in patients with CVID, as well as XLA, both primary immune defects marked by a profound loss of humoral immunity. Bacterial translocation in CVID has previously been hypothesized, though not proven, based on indirect markers of cellular activation. Perreau et al. reported that bacteria-specific CD4^+^T cells from patients with CVID expressed higher levels of PD-1, suggesting chronic bacterial antigen exposure (27). Jorgensen et al. noted soluble CD25 in plasma of patients with CVID with altered gut microbiota (43). However, direct evaluations, such as measuring circulating LPS (a component of Gram-negative bacterial cell wall), have yielded contradictory results in CVID, with detection of LPS/endotoxin in some patients with low serum IgG (27, 28); other studies have showed no differences in circulating LPS levels between patients with CVID and controls using the same assay (29, 30). Here, in a large cohort of 92 patients with CVID, we also did not detect circulating endotoxin/LPS using 4 commercial assays. Given that 90 out of 92 patients with CVID were on Ig replacement therapy, this finding may reflect the rapid binding of endotoxin/LPS by infused IgG antibody (27, 44, 45). In this cohort, all patients with replaced IgG had serum IgG levels (mean 999 mg/dL) well above a level previously suggested as sufficient for reducing circulating endotoxins (490 mg/dL) (27). It is also possible that infused IgG in patients interferes with the endotoxin/LPS detection assay, which we also demonstrated here in vitro. However, by measuring conserved bacterial 16S rDNA in the serum, we were able to circumvent these limitations, and we established the existence of bacterial translocation from the gut to circulation in patients with CVID and in patients with XLA.

In CVID, detection of significantly elevated circulating bacterial DNA was accompanied by elevated measures of systemic immune activation: serum LBP and sCD14. Our results are consistent with prior reports noting increased sCD14 in smaller CVID cohorts (29, 30) and in other disease states characterized by the presence of bacterial translocation (46, 47). Soluble CD14, in particular, may be released in the setting of exposure to various microbial products, including LPS (Gram-negative bacteria), Gram-positive bacteria components, and CpG DNA (23–26, 48). Consistent with these findings, we observed a positive correlation between sCD14 and serum 16S rDNA levels, suggesting that its elevation reflects the translocated bacterial DNA as an immune stimulant in this disorder.

Further evidence of a specific gastrointestinal barrier defect in CVID was identified with elevated serum levels of both zonulin and I-FABP. As known markers of gut epithelial integrity, increased serum zonulin and I-FABP have similarly been found in celiac disease, inflammatory bowel disease, HIV, and other chronic inflammatory diseases (47, 49–52). While specificity of the current commercial zonulin assay has been a topic of debate (31, 53), zonulin levels have been correlated with experimental and clinical parameters of intestinal permeability (51). Our results suggest that the combination of humoral immune defects and intestinal barrier dysfunction underlie the bacterial translocation phenomenon in CVID. However, the resulting inflammatory state in CVID may further promote barrier defects, creating a vicious cycle of bacterial translocation and immune stimulation (54, 55).

In addition to being associated with markers of immune activation, we showed that high circulating bacterial DNA levels were strongly associated clinically with the inflammatory CVID phenotype in this large cohort. In a similar manner, bacterial translocation has been implicated in a number of chronic inflammatory states, including HIV infection, cirrhosis, and inflammatory bowel diseases, among others (47, 56, 57). Our finding extends earlier observations that altered gut microbiota was associated with markers of immune activation (sCD14, sCD25) in CVID (30, 43). With evidence of mucosal barrier dysfunction (elevated zonulin and I-FABP levels), continuous absorption of various bacterial products, including DNA, would likely activate systemic immune responses through recognition by innate pattern recognition receptors (58) with complex immunological consequences.

Focusing on the IFN signature that characterizes the inflammatory state in CVID (10), we found that patients with high circulating bacterial DNA also had high same-day serum IFN-γ in vivo. We further demonstrated bacterial DNA–induced IFN-γ secretion in both CVID and control PBMCs in vitro. Our findings are consistent with prior observations on the capability of bacterial DNA to induce host immune responses in experimental models (59–61). These results support the hypothesis that the translocated bacteria DNA is bioactive and capable of driving inflammatory complications in CVID. Interestingly, bacterial DNA induced significantly higher IFN-γ secretion in patients with CVID with inflammatory complications in comparison to those without complications and healthy controls, suggesting that their cells were primed to respond. Taken together, these findings indicate that it is not the mere presence of an inflammatory stimulus that determines pathology, but that the host’s response to such a stimulus likely plays a determining role.

Patients with CVID with severe reduction of isotype-switched memory B cells had significantly elevated circulating 16S rDNA levels. The loss of these cells has previously been associated with reduced IgA, IgG, and specific antibody responses, as well as higher risks for inflammatory complications (41, 62–64). In line with previous observations that IgG replacement alone does not appear to ameliorate most inflammatory complications in CVID (5, 6), the translocation of bacterial DNA in these patients with CVID was unaffected by IgG replacement therapy. Taken together, these data indicate that severe defects in the B cell compartment are likely related to defects in host-commensal compartmentalization in CVID. Additionally, while IgG replacement may bind or neutralize some bacterial products (e.g., endotoxin), it appeared insufficient to abolish all facets of bacterial translocation. While loss of secretory antibodies (e.g., intestinal IgA) may play a determining role, serum IgA was very low or absent in patients in this study because of their diagnosis.

Interestingly, although elevated circulating bacterial DNA was observed in CVID and in patients with XLA, there was a notable differential host response to microbial stimulus between these 2 groups, with lack of prominent systemic immune activation in XLA (no significant elevations of LBP or sCD14). This finding is consistent with the clinical phenotype of XLA, in that these patients generally lack the typical inflammatory complications seen in CVID (65). XLA results from a B cell differentiation arrest due to mutations in the BTK gene (66, 67), but the additional loss of functional BTK in monocytes, macrophages, neutrophils, and DCs in these patients could blunt or eliminate some of the inflammatory responses. Since BTK is involved in multiple pattern recognition receptor signaling pathways (e.g., TLRs) (68, 69), the absence of BTK may also dampen the immune response to translocated microbes/microbial products. Indeed, bacterial DNA–induced cytokine production has been shown to be impaired in PBMCs derived from patients with XLA (69). Here, we found a differential bacterial DNA–induced IFN-γ response between patients with CVID and patients with XLA, but this did not appear to be a function of B cells alone because there was no significant correlation between peripheral B cell numbers and serum IFN-γ levels in vivo among patients with CVID. Given that BTK inhibition resulted in reduced IFN-γ responses to bacterial DNA stimuli among patients with CVID and inflammatory complications in vitro, we suggest that BTK is a host modifier in immune responses to microbial translocation. It is also possible that a more intact gut barrier integrity in XLA, as suggested by the relatively normal zonulin and I-FABP levels here, contribute to this differential outcome. Further investigation of the molecular and cellular basis of these differential immune responses in CVID and XLA could be revealing and potentially of therapeutic importance.

By performing 16S rRNA gene profiling, we identified circulating bacterial DNA from a diverse range of commensals, rather than select bacterial species, in patients with CVID. Even though the gut is almost certainly the predominant source of bacterial translocation, we cannot exclude this occurrence from other mucosal sites (e.g., respiratory tract). In the CVID sera, we identified an abundance of *Lachnospiraceae, Ruminococcaceae, Erysipelotrichaceae,* and *Bacteroidaceae* DNA — all gut commensal families with members previously reported to be well-coated by secretory IgA in healthy humans (37–39). We also identified an abundance of *Comamonadaceae* DNA, which was previously found to be highly coated with IgM in people with selective IgA deficiency (40). Jorgensen et al. found an increased abundance of the genera *Roseburia* (part of the *Lachnospiraceae* family) and *Bacilli* (*Bacillaceae*) in the gut of patients with CVID relative to healthy individuals (43); consistent with this prior finding, we observed that *Roseburia* (*Lachnospiraceae*) and *Bacilli* (*Bacillaceae*) DNA were highly abundant in CVID sera. In contrast, our serum analysis did not detect bacteria DNA from the family *Enterobacteriaceae*, which was reported to be relatively increased in the gut of patients with CVID compared with healthy controls (70). Lastly, through transkingdom network analysis of the duodenal microbiome in patients with CVID with or without enteropathy, *Acinetobacter baumannii* has previously been proposed to be a candidate inciting organism in patients with CVID with enteropathy (71); the DNA of this species was, however, not detected in the sera of patients with CVID in this report regardless of the presence or absence of enteropathy.

There are limitations to this study. We detected elevated circulating bacterial product (16S rDNA of various gut taxa) in patients with CVID in the absence of overt bacteremia (i.e., demonstrated growth of intact bacteria through conventional blood cultures), but the lack of direct access to mesenteric vein/lymph nodes in our human study prevented us from more conclusively determining whether there was also the translocation of some intact bacteria through the gut epithelium in CVID. In addition, although bacterial 16S rDNA has been used to detect bacterial translocation in other fields, its precise form(s) in the circulation remain to be fully elucidated. Future dedicated structural-based studies on the biochemical forms of the translocated bacterial DNA and whether it may be bound to additional bacterial debris may also be revealing.

In summary, we showed that bacterial translocation in patients with profound primary antibody defects leads to circulating bioactive bacterial DNA capable of immune activation and is associated with IFN-γ–linked inflammatory manifestations in CVID. These data established a mechanism for immune activation in CVID and identified bacterial DNA translocation, barrier dysfunction, and the ensuing host response as interventional targets to modify the clinical outcomes of CVID. Future studies of host-commensal immune regulation in this and other human primary immunodeficiency disorders should improve our understanding of this complex relationship and therapeutic approaches.

## Methods

### Patients and serum.

All participants recruited for this study were patients at the Mount Sinai Clinical Immunology Faculty Practice. Serum from patients with CVID (1, 2), patients with XLA (1), and healthy controls were drawn into 6 mL gold top rubber-sealed sterile Vacutainer SST II tubes (BD Diagnostics). Baseline patient characteristics are summarized in Table 1. In the CVID cohort, autoimmunity/inflammatory complications included lymphoid hyperplasia/splenomegaly (*n* = 52), hematologic autoimmunity (autoimmune hemolytic anemia and/or immune thrombocytopenic purpura, *n* = 38), chronic lung disease (interstitial lung disease and/or granulomatous lung disease, *n* = 35), enteropathy (*n* = 33), and granulomas (*n* = 26). The tubes were maintained in the upright position until the serum was aseptically removed in a sterile hood for bacterial and other assays.

### Quantitative PCR for bacterial 16S rDNA.

Quantitative PCR was performed blinded for clinical characteristics. The amplification reaction mixture was composed of 10 μL of LightCycler 480 SYBR Green (Roche), 1 μL of 16S bacterial rDNA forward primer (5′-AAC AGG ATT AGA TAC CCT GGT AG-3′, 1 μL of reverse primer (5′-GGT TCT KCG CGT TGC WTC-3′) (72), 1 μL of dsDNAase (Thermo Fisher Scientific), 1 μL of 10× dsDNAase buffer (Thermo Fisher Scientific), and 5 μL of SYBR Green reaction mix (Roche). Then, 19 μL of the reaction mixture was added to each well of a 384-qPCR plate, followed by 1 μL of DNA isolated from 200 μL of serum (DNeasy Blood and Tissue Kit, Qiagen) or the *E*. *coli* bacterial DNA standard. The DNA was amplified in triplicate, and mean values were calculated. The reaction conditions for amplification of DNA were 95°C for 10 minutes, followed by 45 cycles at 95°C for 10 seconds, 60°C for 20 seconds, and 72°C for 5 seconds. The bacterial DNA standard was prepared from *E*. *coli*–competent cells (Thermo Fisher Scientific) using DNeasy Blood and Tissue Kit (Qiagen) and concentrations were measured by NanoDrop (Thermo Fisher Scientific). Using the *E*. *coli* genome length (4,700,000 bp), the number of *E*. *coli* DNA copies was calculated (https://cels.uri.edu/gsc/cndna.html) and serial dilutions of this standard were used to define DNA copy numbers in a standard curve.

### Serum LBP, sCD14, zonulin, I-FABP, and IFN-γ assays.

Concentrations of sCD14, LBP, zonulin, and I-FABP were determined in serum (with dilutions of 1:2500, 1:1000, 1:10, 1:10, respectively) using separate immunoassays (R&D Systems). Serum IFN-γ, diluted 1:2, from patients not on a systemic immunosuppressant was determined using a human IFN-γ ELISA (R&D Systems). FLUOstar Omega multimode microplate reader (BMG Labtech) was employed. Representative data from duplicate experiments were presented.

### Endotoxin/LPS assays.

The endotoxin detection assays used were EndoLISA (Hyglos), Pierce LAL Chromogenic Endotoxin Quantitation kit (Thermo Fisher Scientific), Limulus Amebocyte Lysate Chromogenic Endpoint Assay (Hycult Biotech), and ToxinSensor (GenScript). To examine potential interference by Ig, bacterial endotoxin (Invivogen) was coated on ELISA plates (Thermo Fisher Scientific); subsequently, 0.003 to 11.4 mg/mL of a commercial polyclonal Ig was added and left to bind for 2 hours at room temperature. Detection of endotoxin-bound commercial Ig was carried out with HRP-conjugated anti–human IgG antibody (Southern Biotech).

### Flow cytometry.

Four-color flow cytometry with LSRII cytometer and FacsDIVA software (BD Biosciences) was performed on patient PBMCs using anti–human mAbs CD19 PC5 (Beckman Coulter, IM2643U), CD27 FITC (Dako, F7178), IgM APC (Jackson ImmunoResearch, 309605095), and IgD PE (BD Pharmingen, 555779). Analysis was performed with FlowJo software.

### DNA extraction, 16S rDNA amplification, and multiplex sequencing.

DNA was extracted from collected 25 CVID serum samples following handling guidelines for microbiome studies as previously described (73). Briefly, collected serum samples were introduced into a biosafety cabinet that was decontaminated prior to introduction of the samples by UV treatment for 1 hour and subsequently decontaminated with 5% bleach. Personnel handling the samples were wearing isolation gowns, clean gloves that were sprayed beforehand with 5% bleach and 70% ethanol, and a face mask. DNA was extracted using the DNeasy UltraClean Microbial Kit (Qiagen), including 12 blank-extraction controls. Amplicon preparation and sequencing were performed as previously described (74). Briefly, bacterial 16S rDNA PCR, including 25 no-template PCR controls, was set up in a separate PCR workstation using dual-indexed primers. PCR reactions contained 1 μM for each primer, 4 ng DNA, and Phusion Flash High-Fidelity PCR Master Mix (Thermo Fisher Scientific). Reactions were held at 98°C for 30 seconds, proceeding to 50 cycles at 98°C for 10 seconds, 45°C for 30 seconds, and 72°C for 30 seconds and a final extension of 2 minutes at 72°C. Amplicons were evaluated by gel electrophoresis. The sequencing library was prepared by combining equivolume amounts of each amplicon, size-selected and concentrated using AMPure XP beads (0.8×, Beckman Coulter). Library concentration was quantified by Qubit (Thermo Fisher Scientific) and qPCR, mixed with 20% PhiX, diluted to 4 pM, and subjected to paired-end sequencing (Reagent Kit V2, 2 × 150 bp) on an Illumina MiSeq sequencer.

Resulting FASTQ files were analyzed using the QIIME2 version 2019.10 (75) and the DADA2 (76) denoised-paired plugin with a truncation length of 150 bp and 145 bp for the forward and reverse read, respectively. Amplicon sequence variants (ASVs) were classified using the Scikit-Learn plugin (77) using the gg-13-8-99-515-806-nb classifier. Resulting ASV tables were filtered using a minimum depth of 1000 and a minimal ASV frequency of 30, and any ASVs observed in the blank extraction and nontemplate PCR controls were filtered out. Taxonomic plots were generated from the filtered ASV tables using R/ggplot2 (78).

### Bacterial DNA and PBMC coculture.

Bacterial DNA was prepared from *E*. *coli*–competent cells (Thermo Fisher Scientific) using DNeasy Blood and Tissue Kit (Qiagen). PBMCs freshly isolated from each study participant (5 × 10^5^ cells) were cocultured with *E*. *coli* bacterial DNA (0 to 3 × 10^4^ DNA copies [7.5 pg/mL]). Coculture supernatants were collected and analyzed on day 3. IFN-γ levels in the supernatants were measured using a human IFN-γ ELISA (R&D Systems) and the FLUOstar Omega multimode microplate reader (BMG Labtech). For bacterial DNA digestion, *E*. *coli* bacterial DNA was incubated with DNase I (Thermo Fisher Scientific) at 37°C for 15 minutes. For BTK inhibition, PCI 29732 (final concentration 0.5 nM, Tocris Bioscience) (42) was added to PBMC cultures at 2, 8, and 12 hours prior to the addition of *E*. *coli* bacterial DNA (3 × 10^4^ copies, 7.5 pg/mL). To examine potential interference by human cell-free DNA, an equivalent concentration (7.5 pg/mL) of human genomic DNA (Promega) was added to the bacterial DNA and PBMC coculture, and IFN-γ output was measured as described previously.

### Statistics.

Statistical differences for 2-group comparisons were analyzed using an 2-tailed, unpaired *t* test for normally distributed data and a Mann-Whitney *U* test for nonnormal data. Multiple group comparisons were analyzed using 1-way ANOVA with Tukey’s multiple-comparison test for normally distributed data and Kruskal-Wallis test with Dunn’s multiple-comparison test for nonnormal data. Bivariate correlations between variables were calculated using Spearman’s test. All reported *P* values were 2-sided, and *P* values less than 0.05 were considered significant. All calculations were performed using GraphPad Prism software.

### Study approval.

This study was approved by the IRB of the Icahn School of Medicine at Mount Sinai and was carried out in accordance with the Code of Ethics of the World Medical Association (Declaration of Helsinki). Written informed consent was received from participants prior to inclusion in the study.

## Author contributions

HH and CC conceived the project, designed and performed research, analyzed and discussed data, prepared the figures, and wrote the manuscript. LR and GB performed experiments and analyzed and discussed data. AE performed experiments and discussed data. All authors contributed to critical revision of the manuscript and approved the final draft.

## Supplementary Material

Supplemental data

## Figures and Tables

**Table 1:**
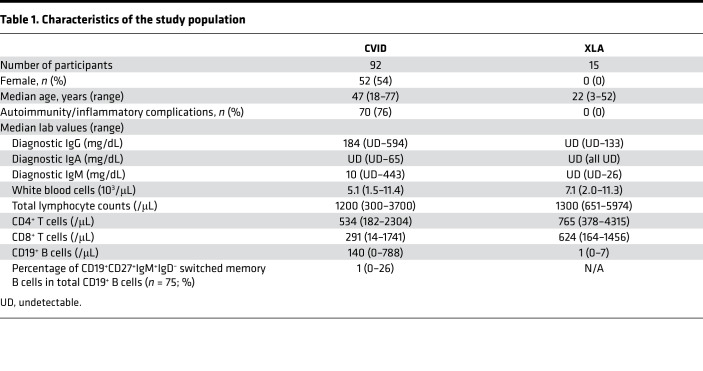
Characteristics of the study population

## Version 1 10/08/2021

Electronic publication
