# Supplementary material for: Circulating bioactive bacterial DNA is associated with immune activation and complications in common variable immunodeficiency
Source: JCI Insight. 2021 Oct 8;6(19):e144777. doi: 10.1172/jci.insight.144777 (PMC8525635; doi:10.1172/jci.insight.144777)
Supplement: Supplemental data [file jciinsight-6-144777-s263.pdf]

Supplemental data

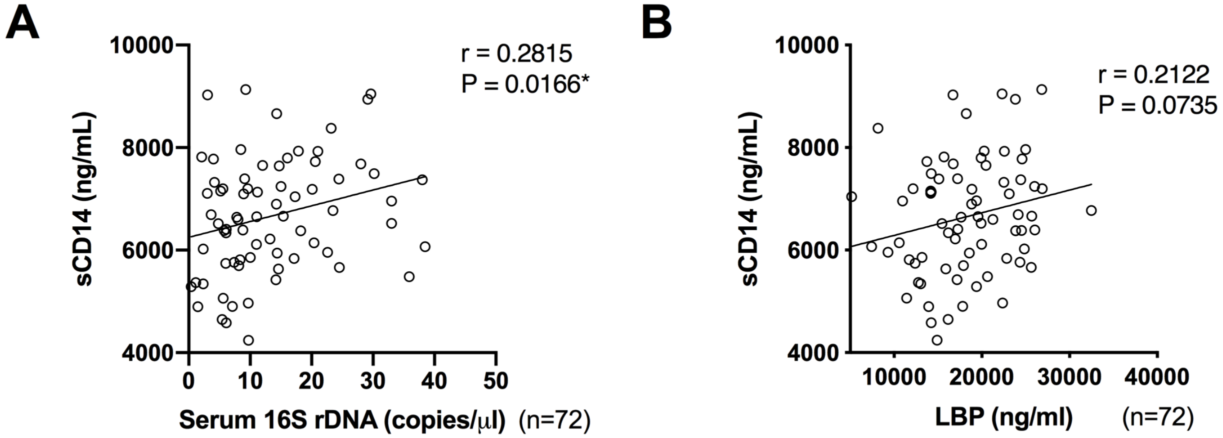

Fig. S1. Serum sCD14 correlates with 16s rDNA levels in CVID sera. Correlation (Spearman) between **(A)** sCD14 and 16S rDNA, and **(B)** sCD14 and LBP in CVID sera. *P < 0.05.

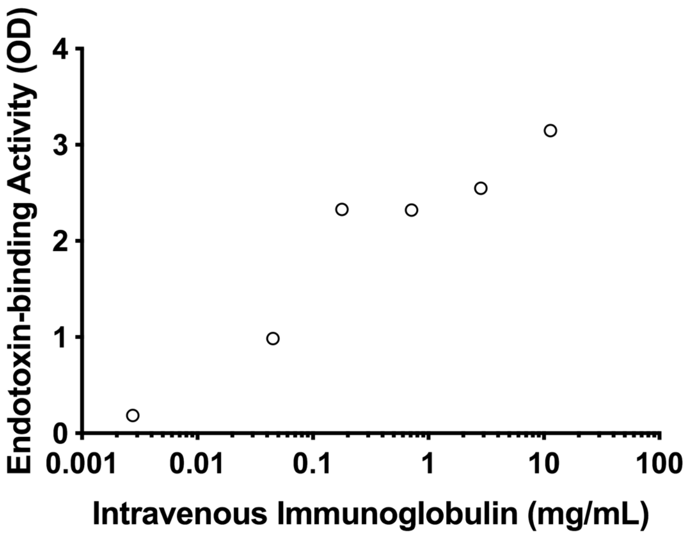

Fig. S2. Dose-dependent IgG endotoxin-binding activity of intravenous immunoglobulin. Endotoxin-binding IgG detected by HRP-conjugated anti-human IgG, y axis. OD, optical density.

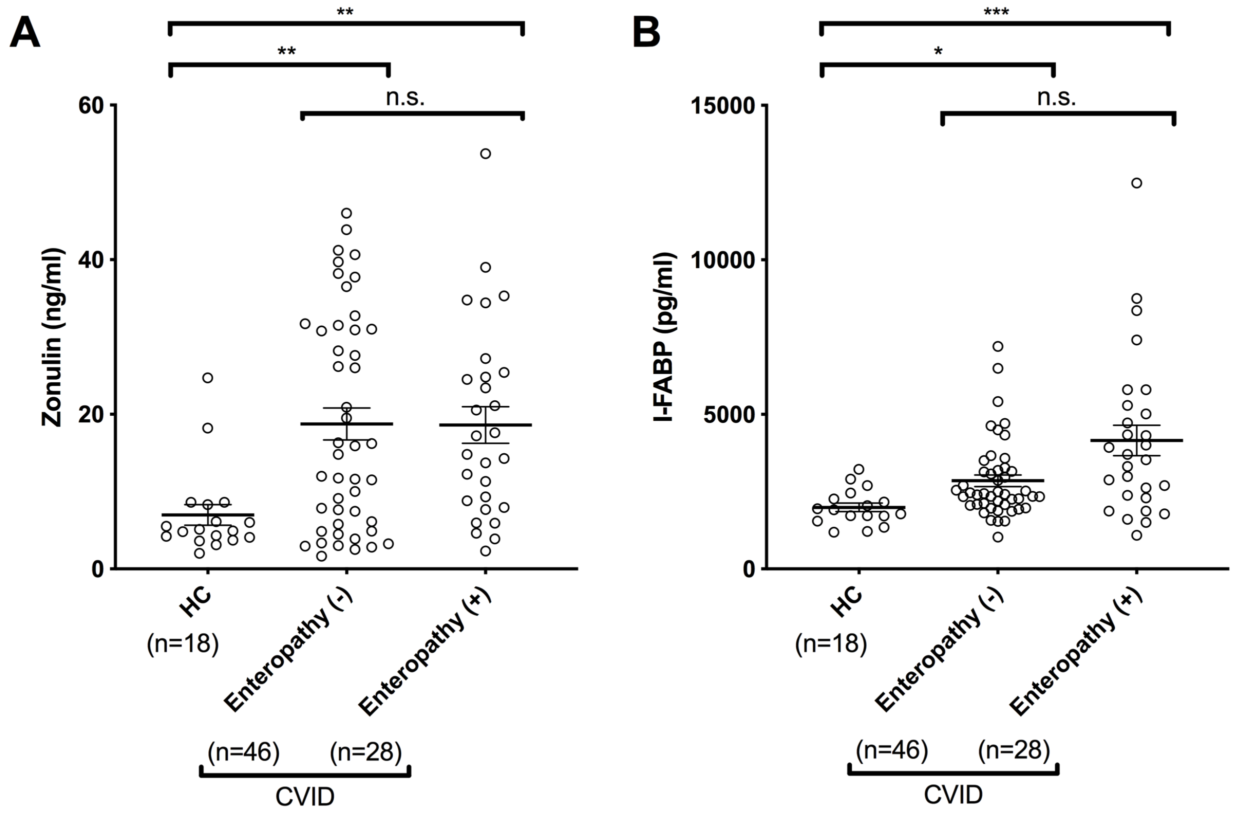

Fig. S3. Gut barrier dysfunction is present in CVID with or without clinical enteropathy. **(A)** Serum zonulin and **(B)** I-FABP levels in HC and CVID subjects with or without clinically-overt enteropathy. The data are expressed as the mean ± SEM. * $P < 0.05$, ** $P < 0.01$, *** $P < 0.001$ by Kruskal-Wallis with Dunn's multiple comparison post hoc test. n.s., not significant.

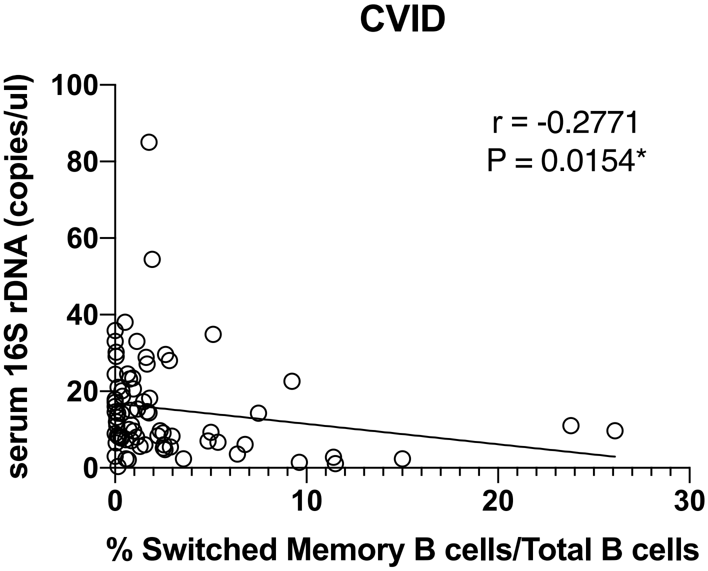

Fig. S4. Serum 16S rDNA levels negatively correlated (Spearman) with the percentages of circulating switched memory B cells (CD19+CD27+IgM-IgD- switched memory B cells/CD19+ B cells) in CVID.

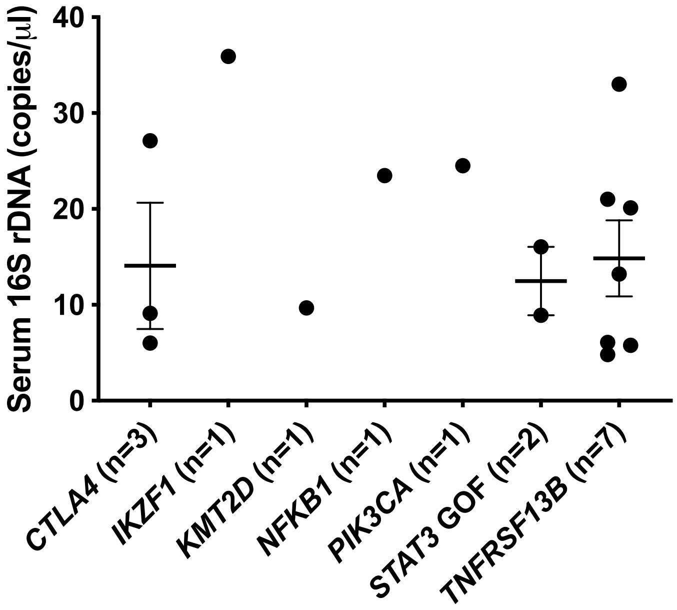

Fig. S5. Serum 16S rDNA levels in CVID subjects with a known monogenic defect. *CTLA4*, cytotoxic T-lymphocyte-associated protein 4; *IKZF1*, IKAROS family zinc finger 1; *KMT2D*, lysine methyltransferase 2D; *NFKB1*, nuclear factor kappa-light-chain-enhancer of activated B cells 1; *PIK3CA*, phosphoinositide 3-kinase; *STAT3* GOF, signal transducer and activator of transcription 3 gain-of-function; T*NFRSF13B*, tumor necrosis factor receptor superfamily member 13B. The data are expressed as the mean ± SEM.

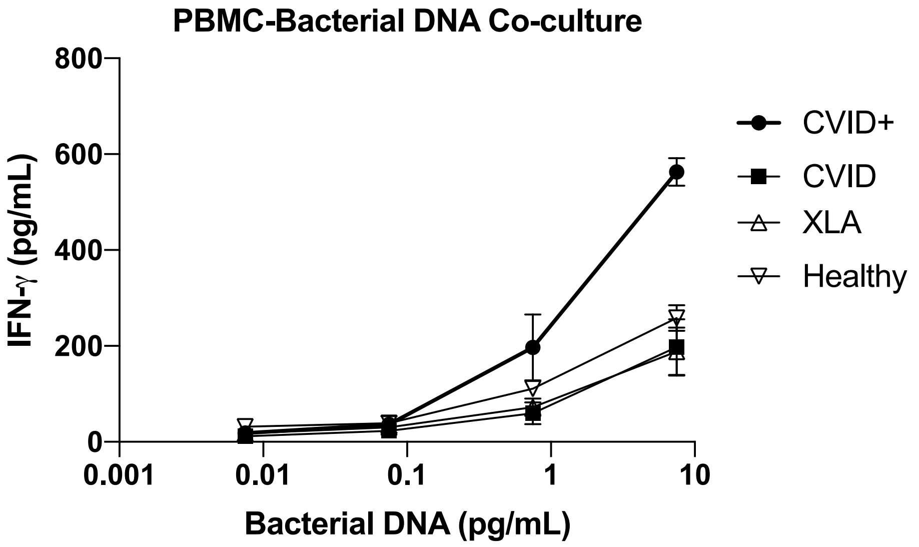

Fig. S6. Dose-dependent IFN-γ response to bacterial DNA co-culture in PBMC isolated from Healthy (n=5), XLA (n=5), CVID (n=4), and CVID+ (inflammatory CVID, n=4) subjects. The data are expressed as the mean ± SEM

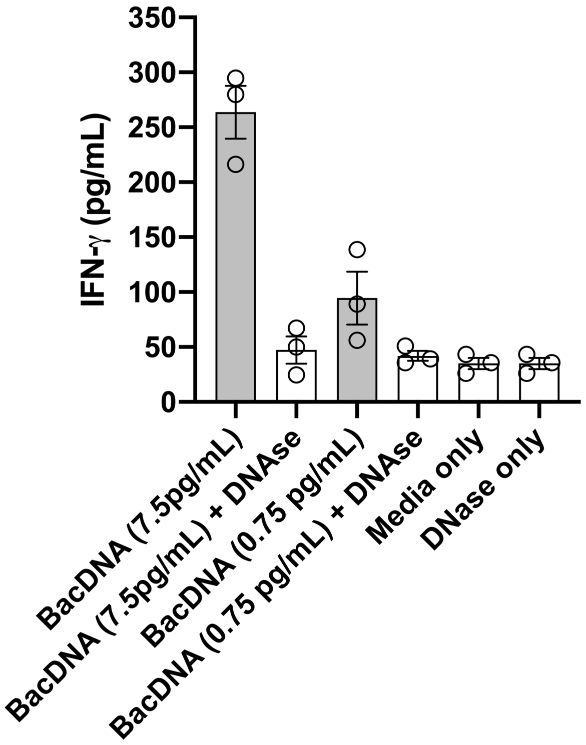

Fig. S7. IFN-γ response to bacterial DNA (BacDNA) co-culture in PBMC isolated from healthy subjects (n=3) with or without the digestion of bacterial DNA by DNAse. The data are expressed as mean ± SEM.

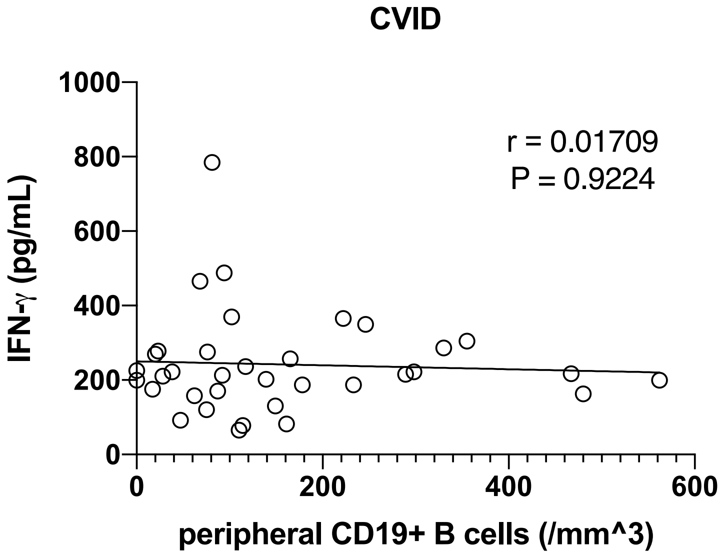

Fig. S8. The lack of correlation (Spearman) between serum IFN-γ and peripheral CD19+ B cells in CVID subjects.

k__Bacteria;p__Firmicutes;c__Clostridia;o__Clostridiales;f__Lachnospiraceae [Roseburia, Blautia, Dorea]
k__Bacteria;p__Proteobacteria;c__Betaproteobacteria;o__Burkholderiales;f__Comamonadaceae
k__Bacteria;p__Firmicutes;c__Clostridia;o__Clostridiales;f__Ruminococcaceae
k__Bacteria;p__Proteobacteria;c__Alphaproteobacteria;o__Sphingomonadales;f__Sphingomonadaceae
k__Bacteria;p__Firmicutes;c__Bacilli;o__Bacillales;f__Bacillaceae [Bacillus, Lactobacillus]
k__Bacteria;p__Firmicutes;c__Erysipelotrichi;o__Erysipelotrichales;f__Erysipelotrichaceae
k__Bacteria;p__Firmicutes;c__Bacilli;o__Lactobacillales;f__Streptococcaceae
k__Bacteria;p__Actinobacteria;c__Actinobacteria;o__Bifidobacteriales;f__Bifidobacteriaceae
k__Bacteria;p__Firmicutes;c__Clostridia;o__Clostridiales;f__Veillonellaceae
k__Bacteria;p__Bacteroidetes;c__Flavobacteriia;o__Flavobacteriales;f__Flavobacteriaceae
k__Bacteria;p__Actinobacteria;c__Actinobacteria;o__Actinomycetales;f__Brevibacteriaceae
k__Bacteria;p__Firmicutes;c__Bacilli;o__Lactobacillales;f__Lactobacillaceae
k__Bacteria;p__Bacteroidetes;c__Bacteroidia;o__Bacteroidales;f__Bacteroidaceae
k__Bacteria;p__Firmicutes;c__Bacilli;o__Lactobacillales;f__Leuconostocaceae
k__Bacteria;p__Actinobacteria;c__Actinobacteria;o__Actinomycetales;f__Microbacteriaceae
k__Bacteria;p__Firmicutes;c__Bacilli;o__Bacillales;f__[Thermicanaceae]
k__Bacteria;p__Verrucomicrobia;c__Verrucomicrobiae;o__Verrucomicrobiales;f__Verrucomicrobiaceae
k__Bacteria;p__Proteobacteria;c__Betaproteobacteria;o__Burkholderiales;f__Oxalobacteraceae
k__Bacteria;p__Actinobacteria;c__Actinobacteria;o__Actinomycetales;f__Micrococcaceae
k__Bacteria;p__Firmicutes;c__Clostridia;o__Clostridiales;f__Peptostreptococcaceae
k__Bacteria;p__Firmicutes;c__Clostridia;o__Clostridiales;f__[Tissierellaceae]
k__Bacteria;p__Firmicutes;c__Bacilli;o__Bacillales;f__Staphylococcaceae
k__Bacteria;p__Bacteroidetes;c__Bacteroidia;o__Bacteroidales;f__Prevotellaceae
k__Bacteria;p__Proteobacteria;c__Betaproteobacteria;o__Burkholderiales;f__Alcaligenaceae
k__Bacteria;p__[Thermi];c__Deinococci;o__Deinococcales;f__Deinococcaceae
k__Bacteria;p__Proteobacteria;c__Gammaproteobacteria;o__Pseudomonadales;f__Pseudomonadaceae
k__Bacteria;p__Firmicutes;c__Bacilli;o__Bacillales;f__[Exiguobacteraceae]
k__Bacteria;p__Actinobacteria;c__Actinobacteria;o__Actinomycetales;f__Actinomycetaceae
k__Bacteria;p__Bacteroidetes;c__Flavobacteriia;o__Flavobacteriales;f__Cryomorphaceae
k__Bacteria;p__Actinobacteria;c__Actinobacteria;o__Actinomycetales;f__Mycobacteriaceae
k__Bacteria;p__Proteobacteria;c__Betaproteobacteria;o__Burkholderiales;f__Burkholderiaceae
k__Bacteria;p__Firmicutes;c__Clostridia;o__Clostridiales;f__Clostridiaceae
k__Bacteria;p__Proteobacteria;c__Gammaproteobacteria;o__Pseudomonadales;f__Moraxellaceae
k__Bacteria;p__Actinobacteria;c__Coriobacteriia;o__Coriobacteriales;f__Coriobacteriaceae
k__Bacteria;p__Bacteroidetes;c__[Saprospirae];o__[Saprospirales];f__Saprospiraceae
k__Bacteria;p__Actinobacteria;c__Actinobacteria;o__Actinomycetales;f__Dermabacteraceae
k__Bacteria;p__Bacteroidetes;c__Bacteroidia;o__Bacteroidales;f__[Barnesiellaceae]

k__Bacteria;p__Actinobacteria;c__Actinobacteria;o__Actinomycetales;f__Intrasporangiaceae
k__Bacteria;p__Bacteroidetes;c__Bacteroidia;o__Bacteroidales;f__[Odoribacteraceae]
k__Bacteria;p__Proteobacteria;c__Alphaproteobacteria;o__Rhizobiales;f__Phyllobacteriaceae
k__Bacteria;p__Proteobacteria;c__Alphaproteobacteria;o__Caulobacterales;f__Caulobacteraceae
k__Bacteria;p__Spirochaetes;c__[Leptospirae];o__[Leptospirales];f__Leptospiraceae
k__Bacteria;p__Proteobacteria;c__Gammaproteobacteria;o__Aeromonadales;f__Aeromonadaceae
k__Bacteria;p__Firmicutes;c__Bacilli;o__Turicibacterales;f__Turicibacteraceae
k__Bacteria;p__Proteobacteria;c__Alphaproteobacteria;o__Rhodospirillales;f__Rhodospirillaceae
k__Bacteria;p__Bacteroidetes;c__[Saprospirae];o__[Saprospirales];f__Chitinophagaceae

Table S1. Taxonomic profile of translocated bacteria in CVID ranked by abundance.
